# Machine Learning Incorporating Host Factors for Predicting Survival in Head and Neck Squamous Cell Carcinoma Patients

**DOI:** 10.3390/cancers13184559

**Published:** 2021-09-11

**Authors:** Han Yu, Sung Jun Ma, Mark Farrugia, Austin J. Iovoli, Kimberly E. Wooten, Vishal Gupta, Ryan P. McSpadden, Moni A. Kuriakose, Michael R. Markiewicz, Jon M. Chan, Wesley L. Hicks, Mary E. Platek, Anurag K. Singh

**Affiliations:** 1 Department of Biostatistics and Bioinformatics, Roswell Park Comprehensive Cancer Center, Elm and Carlton Streets, Buffalo, NY 14263, USA; Han.Yu@roswellpark.org; 2 Department of Radiation Medicine, Roswell Park Comprehensive Cancer Center, 665 Elm Street, Buffalo, NY 14203, USA; SungJun.Ma@RoswellPark.org (S.J.M.); Mark.Farrugia@roswellpark.org (M.F.); Austin.Iovoli@RoswellPark.org (A.J.I.); Mary.Platek@RoswellPark.org (M.E.P.); 3 Jacobs School of Medicine and Biomedical Sciences, University at Buffalo, The State University of New York, 955 Main Street, Buffalo, NY 14203, USA; 4 Department of Head and Neck Surgery, Roswell Park Comprehensive Cancer Center, 665 Elm Street, Buffalo, NY 14203, USA; Kimberly.Wooten@RoswellPark.org (K.E.W.); Vishal.Gupta@RoswellPark.org (V.G.); Ryan.McSpadden@RoswellPark.org (R.P.M.); Moni.Kuriakose@RoswellPark.org (M.A.K.); Michael.Markiewicz@RoswellPark.org (M.R.M.); Jon.Chan@RoswellPark.org (J.M.C.); Wesley.Hicks@RoswellPark.org (W.L.H.J.); 5 Department of Oral and Maxillofacial Surgery, School of Dental Medicine, University at Buffalo, The State University of New York, 3435 Main Street, Buffalo, NY 14214, USA; 6 Department of Neurosurgery, Department of Surgery, Jacobs School of Medicine and Biomedical Sciences, University at Buffalo, The State University of New York, 955 Main Street, Buffalo, NY 14203, USA; 7 Department of Cancer Prevention and Control, Roswell Park Comprehensive Cancer Center, 665 Elm Street, Buffalo, NY 14203, USA; 8 Department of Dietetics, D’Youville College, 270 Porter Avenue, Buffalo, NY 14201, USA

**Keywords:** overall survival, random survival forest, stratification, head and neck neoplasms

## Abstract

**Simple Summary:**

Among head and neck squamous cell carcinoma patients, the five-year survival rates have seen little improvement over the past decade. Prediction of a cancer patient’s clinical outcome is challenging but important for patient counseling and treatment planning. In this work, we evaluated common machine learning models in predicting head and neck squamous cell carcinoma patients’ overall survival based on clinical, demographic features and host factors. We identified the top-performing model and verified host factors can improve the model performance when proper methods are applied. The findings are of critical importance for improved risk stratification of head and neck squamous cell carcinoma patients and provide targeted supportive care for patients who are likely to have the worst outcome.

**Abstract:**

Prognostication for cancer patients is integral for patient counseling and treatment planning, yet providing accurate prediction can be challenging using existing patient-specific clinical indicators and host factors. In this work, we evaluated common machine learning models in predicting head and neck squamous cell carcinoma (HNSCC) patients’ overall survival based on demographic, clinical features and host factors. We found random survival forest had best performance among the models evaluated, which achieved a C-index of 0.729 and AUROC of 0.792 in predicting two-year overall survival. In addition, we verified that host factors are independently predictive of HNSCC overall survival, which improved the C-index by a margin of 0.026 and the AUROC by 0.034. Due to the strong correlation among host factors, we showed that proper dimension reduction is an important step before their incorporation into the machine learning models, which provides a host factor score reflecting the patients’ nutrition and inflammation status. The score by itself showed excellent discriminating capacity with the high-risk group having a hazard ratio of 3.76 (1.93–7.32, *p* < 0.0001) over the low-risk group. The hazard ratios were further improved to 7.41 (3.66–14.98, *p* < 0.0001) by the random survival forest model after including demographic and clinical features.

## 1. Introduction

Among patients with head and neck squamous cell carcinoma (HNSCC), 5-year survival rates have seen little improvement over the past decade, and, except for HPV-associated oropharyngeal cancers, remain below 50% for locally advanced disease [1]. The current treatment approach is to treat advanced cancers with multimodal therapies. This approach, however, carries significant complication rates and comorbidities. Studies supporting aggressive adjuvant chemotherapy and RT regimens, for example, argue for the need to recognize those patients that will fail traditional treatment regimens and offer them new treatment paradigms [2,3,4]. Therefore, it would be of clinical utility to discriminate between those patients who may or may not respond well to multimodality therapies, or to identify patients who would benefit from less toxic intervention.

In addition to well-known factors such as performance status and disease stage, prior work has demonstrated that patient-specific variables and host factors also influence HNSCC patient survival. The host factors can reflect the patient’s immune, inflammation and nutritional status. It is therefore also logical to assume the host factors are associated with the host–tumor interaction in an interdependent manner. For example, decreased hemoglobin is a significant contributor to a hypoxic tumor environment [5], and impaired oxygen distribution through anemia can contribute to tumor hypoxia and consequently radio resistance [6,7,8,9]. Furthermore, elevated neutrophils can promote an inflammatory tumor microenvironment that can facilitate several oncologic processes, including suppression of the antitumor immune response [10,11,12,13]. Recent evidence suggests that pretreatment values of neutrophils, monocytes, lymphocytes, hemoglobin and albumin, are independently associated with prognosis in patients with HNSCC [5]. While the findings were important, these factors need to be validated using multivariate methods that consider multiple clinical factors in practicable decision models.

Machine learning tools such as random forests [14] (RF) often show superior performance over linear models in classification and regression tasks. The random survival forest (RSF) has extended application of RF model to censored time-to-event data [15], which is well suited to handle multiple, interrelated factors and potential modifiers. In this study, we used and compared both linear and non-linear machine learning tools to optimize the combination of known and novel predictors and thus maximize the prediction accuracy for patients’ overall survival.

## 2. Materials and Methods

### 2.1. Study Cohort

Using a retrospective single-institution database, we reviewed and identified 591 primary HNSCC patients treated at Roswell Park Comprehensive Cancer Center with definitive or post-operative RT between 2003 and 2017. All patients selected for analysis have completed RT. Institutional review board approval was obtained (EDR 103707).

### 2.2. Machine Learning Models

The RSFs are generalizations of RFs for analyzing time-to-event data, which are tree-based ensemble machine learning techniques. Comparing with linear models, the RF model typically shows higher prediction performance because it naturally handles nonlinear relationships and complex interactions among predictors. For a new patient, the RSF predicts the survival probability at any time point (survival function) within eight years after treatment and the cumulative hazard function. The variable importance was used to quantify the contribution of each independent variable to the model predictions. In addition, we evaluated the performance of the Cox proportional hazards model with Least Absolute Shrinkage and Selection Operator (LASSO) regularization (COX). We further compared the DeepSurv, a recently developed Cox proportional hazards deep learning model [16]. Moreover, we also compared the performance of common machine learning methods for discriminating patient survival at given time points (year 1~5 after treatment). The methods evaluated include logistic regression with LASSO regularization [17], RF for classifications [14], extreme gradient boosting machine (GBM) [18] and artificial neural networks (ANN) [19]. For each time point, we built a separate model using each of these methods.

### 2.3. Performance Metrics

The model prediction performance in terms of overall survival (OS) was evaluated by concordance index (C-index). The C-index is a generalization of area under the curves (AUCs) that accounts for the censored data. It is defined as the proportion of concordant pairs out of the total number of evaluable pairs. A pair is concordant if the subject with higher predicted probability of survival also has longer survival time. We also evaluated the model’s performance in predicting patients’ survival at year 1~5. Specifically, a binary indicator of whether a patient’s survival time is greater than a given time point was used as the outcome. Patients censored before the time point were excluded from this assessment. The receiver operating characteristic (ROC) curves were obtained by comparing the predicted risk against the binary outcome. An optimal cutoff was selected by maximizing the Youden index (sensitivity + specificity − 1) of the ROC at year 2. The sensitivity, specificity, positive predictive value (PPV) and negative predictive value (NPV) were reported. Calibration of the selected model was examined by comparing the observed and predicted probability at year 1~5.

### 2.4. Modeling Strategy

Before any steps of model training, the cohort was randomly split into a training/validation set with 70% of subjects and a test set with 30% of subjects. For hosting factors, the observations with levels outside the range of mean ± 3SD (standard deviations) were trimmed at corresponding limits. Independent variables were standardized to have zero mean and unit variance before model training within each set separately. Missing values were imputed using *k*-nearest neighbors (*k*NN) with *k* = 10. Imputation was performed without involving the outcome variables and strictly within the training, validation and test cohorts. A standard principal component analysis (PCA) was used for dimension reduction in the standardized host factors. The PCA was performed only within the training/validation set. The PC scores of the test cohort were predicted by multiplying the training set loading matrix with the standardized test host factor levels. The standardization was performed based on the means and standard deviations of the training set to simulate the real-world prediction settings. We emphasize that no test data set was used in any model training steps, including pre-processing or unsupervised learning by PCA. The tuning of hyperparameters and model selection was performed using cross-validations (CV). The details can be found in the Supplementary Materials File S1.

### 2.5. Model Interpretation

For RSF, the variable importance (VIMP) will be used to quantify the contribution of each independent variable to the model predictions. VIMP measures the decrease in prediction performance (C-index) for the forest ensemble when a variable is randomly permuted. A large positive VIMP shows that the prediction accuracy of the forest is substantially degraded when this variable is noised-up by permutations. Therefore, a large VIMP indicated a potentially predictive variable. Partial dependence plots (PDPs) were used to show the marginal effect of a predictor on the predicted outcome [15]. The details can be found in the Supplementary Materials.

### 2.6. Statistical Analysis

Spearman’s correlation coefficient was used for the correlative analysis among host factors. Hierarchical clustering was performed based on Euclidean distance and complete linkage. The standard errors of C-index and AUROC were estimated using bootstrap methods with 1000 re-samplings. For cross-validation, the standard errors were estimated by the standard deviations of C-indices across runs. The results are presented by mean (SE) unless otherwise specified. For risk stratification, the survival curves were estimated using Kaplan–Meier product limit estimators. The hazard ratios (HRs) were estimated based on Cox proportional hazards models and the 95% confidence intervals (CIs) were reported. The analyses were conducted using R 4.1.0 and R packages randomForestSRC [20], glmnet [21], xgboost [18], neuralnet [19]. DeepSurv was implemented using PySurvival package [22] under Python 3.7.

## 3. Results

### 3.1. Patient Characteristics

The patient characteristics are summarized in Table 1. Among the study cohort, 62% of the patients received CCRT while 18% received CCRT and surgical treatment. The demographic and clinical features of patients are listed in Table 1 and Table 2. The body mass index (BMI) and Karnofsky performance status were measured prior to the starting of radiation. Ethnicity and diagnosis type were not used as candidate predictors as the study cohort is highly homogeneous regarding these two characteristics. A summary of the host factors measured is shown in Table 2. The event rate is 40% within eight years after treatment. The median follow-up of the cohort is 3.27 years, defined as the observation time for those event-free. The cohort was randomly split into a training/validation set (70%, *n* = 414) and test set (30%, *n* = 177).

### 3.2. Host Factors Organized as Clusters

**Figure 1:**
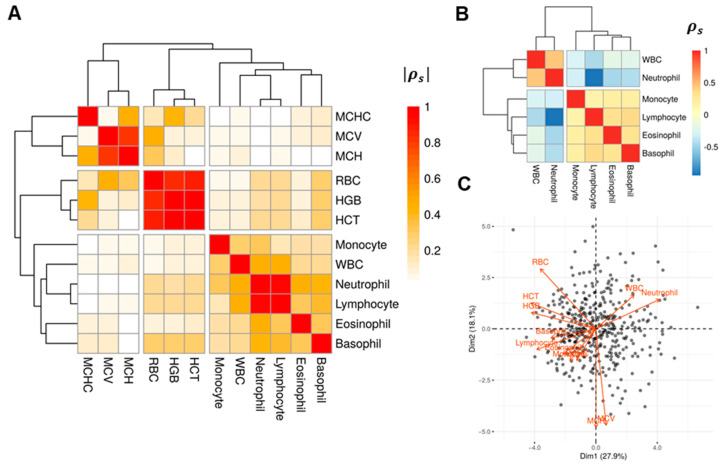
The host factors are mutually correlated. (**A**) The heatmap of the absolute Spearman correlation ($\left|\rho_{s}\right|$) between host factors and the hierarchical clustering based on correlations. The correlations were obtained using the training cohort data. (**B**) The heatmap of the Spearman correlation ($\rho_{s}$) between host factors in the first cluster. (**C**) The biplot of the PCA of the host factors. The biplot shows the projections (contributions) of each host factor to the first two principal components. A large horizontal (vertical) projection suggests the host factor primarily contributes to the PC1 (PC2). Two host factors with projections to the same (opposite) direction suggest they are positively (negatively) correlated with projections onto other PCs ignored. Perpendicular projections imply independence, for example RBC and WBC. The variance explained by each component are shown in corresponding axis labels.

Correlative analyses within the training/validation cohort revealed two major clusters of the host factors. Figure 1A shows the absolute values of the pairwise Spearman’s correlation coefficients among the host factors. Highly correlated factors tend to cluster together and reflect the status of an underlying biological process. A hierarchical clustering shows one mutually correlated group of MCHC, MCV and MCH. A second cluster mainly corresponds to the nutrition status or ability in oxygen delivering. The third cluster primarily reflects the immune or inflammatory status. A closer examination of the third cluster shows the neutrophil and WBC percentages form a strongly correlated group, which is negatively correlated with lymphocyte percentage (Figure 1B). Considering the strong correlations, we performed PCA on the HF variables. Consistent with Figure 1A,B, the biplot (Figure 1C) also shows three groups of variables. The biplot shows the projections (contributions) of each host factor to the first two principal components. A large horizontal (vertical) projection suggests the host factor primarily contributes to the PC1 (PC2). Generally, correlated factors tend to have projections to the same or opposite directions (positively/negatively correlated). The groups with projections in the bottom-left and upper-right quadrants correspond well to the lower cluster in Figure 1A, which reflects inflammatory status. Note that the factors in this cluster also forms two negatively correlated groups, as shown in Figure 1B. The other group in upper-left quadrant corresponds to the bottom cluster in Figure 1A. Although these two groups are mutually independent (projections are perpendicular), the first principal component (PC1) constitutes a composite score of these two domains in opposite directions. We define a host factor (HF) score as HF score = PC1, then a higher HF score corresponds to a higher inflammation and poorer nutrition status.

### 3.3. Model Selection and Evaluation

The COX and RSF models’ performance were compared using the CV within the training/validation set. Adding large number of strongly correlated features is generally harmful to a model’s performance. Therefore, we postulate that including the HF features naively will not provide optimal model performance. Instead, the scores derived from PCA will be naturally uncorrelated summarizing features. We sequentially added the PCs ranked by their eigen values into the model and compared their performance (Table 3). Indeed, adding all HF features naively reduced the performance of RSF and did not maximize COX models’ performance (RSF-ALL/COX-ALL) comparing with the models with clinical/demographic features alone (RSFc/COXc). Using the elastic net or ridge penalties which are better suited for correlated features only showed negligible difference. On the other hand, inclusion of PC scores improved both models’ performance. Overall, the RSF model with only PC1 (HF score) showed best performance. Inclusion of more components does not further improve the validation performance. The COX-1, COX-2 and COX-3 models are identical because PC2 and PC3 were not selected by the model, which further suggests that only PC1 (HF score) is a useful predictor. The superiority of RSF over COX model suggests a nonlinear relationship between predictors and the patient survival or complex interaction among the predictors, which cannot be captured by linear models.

**Figure 2:**
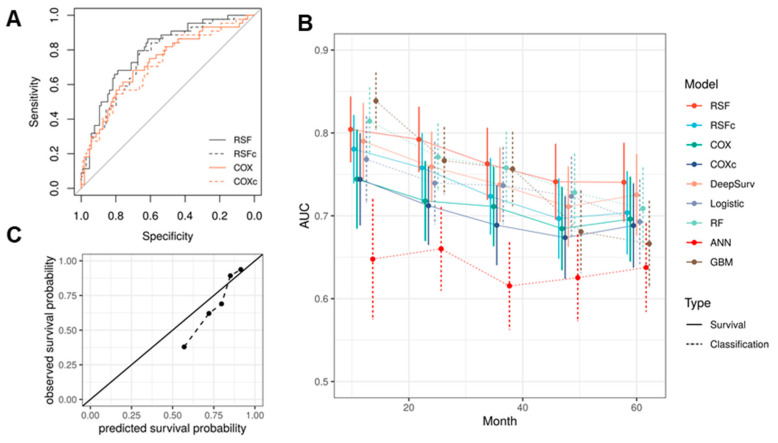
RSF predicts HNSCC patients’ overall survival. (**A**) The ROC curves of the predicted risk by the survival models against observed survival at year two. (**B**) RSF achieved highest AUCs in predicting OS at year 2~5 among the models tested. (**C**) Model calibration of RSF by comparing the predicted and observed two-year survival probability. The black solid line denotes the perfect calibration line.

In the test set, we further compared RSF’s performance with COX model and the RSF model with only clinical/demographic features. Consistent with the CV results, the RSF model performed best in terms of C-index and AUCs (Figure 2A, Table 4). It also showed better performance than DeepSurv. This is not surprising since deep neural network models are often advantageous when sufficient amount of training data available. DeepSurv model is typical successfully applied when there are over thousands of records available for model training [16,23,24,25]. The C-index we obtained using DeepSurv is also close to the result in [23]. Another common strategy for predicting clinical outcome is to treat the prediction of survival at given times points as classification problems. Therefore, performance of the models trained above was further compared with common classification machine learning algorithms in predicting the survival at year 1~5. The methods tested include logistic regression with LASSO regularization (logistic), RF, GBM and ANN. Note that because we are treating the prediction of survival at each time point as separate classification problems, an independent model was trained for each time point in the training/validation set. This is different from RSF, which can predict the survival probability for any time point based on a single model. The result shows that RSF outperforms all classification models in terms of AUC (Figure 2B, Table 4). Only the RF and GBM models showed a better performance at year 1. This is probably because the classification methods cannot fully utilize the time-to-event information. Model calibration of RSF was examined by comparing the predicted and actual two-year survival probability (Figure 2C).

**Figure 3:**
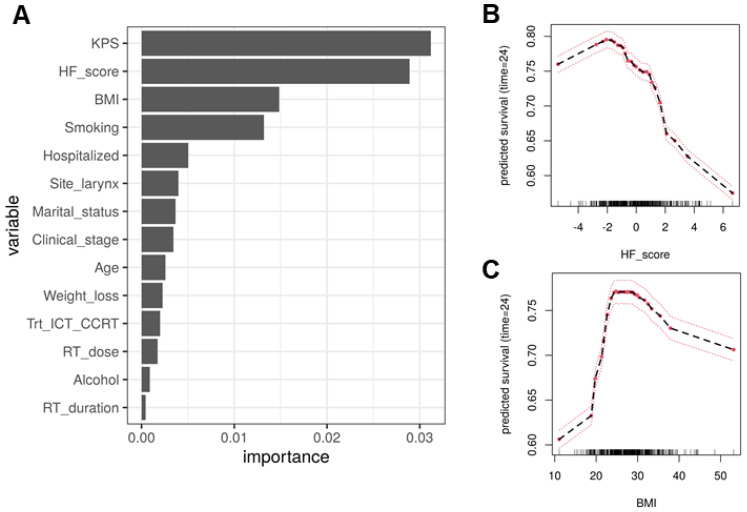
(**A**) Variable importance for the top predictors of the final model. The HF score is among the top ranked variable. (**B**) The partial dependence plot of the BMI with the two-year survival rate as outcome. (**C**) The partial dependence plot of the HF score with the two-year survival rate as outcome.

Variable importance plot shows that the HF score is ranked as one of the top three variables contributing to the prediction (Figure 3A). As a baseline, the variable alone can achieve a C-index of 0.656 (0.034), while the other two top-ranked variables BMI and KPS alone achieve C-indices of 0.648 (0.034) and 0.598 (0.033), respectively.

As shown above, the better performance of RSF over COX model suggest non-linear relationships. This is supported by the partial dependence plots (Figure 3B), which show the relationship between the certain predictors and patients’ two-year survival is not linear or even monotonic. For example, the two-year survival rate increases as BMI increases up to 25, but then plateaus, and even decreases as the BMI further increases. The partial dependence plot shows an overall negative relationship between HF score and two-year survival (Figure 3C), i.e., patients with poorer nutrition and higher inflammation status tend to have worse OS, which is consistent with the previous findings.

### 3.4. Patient Stratification by HF Score and RSF Predicted Risk

**Figure 4:**
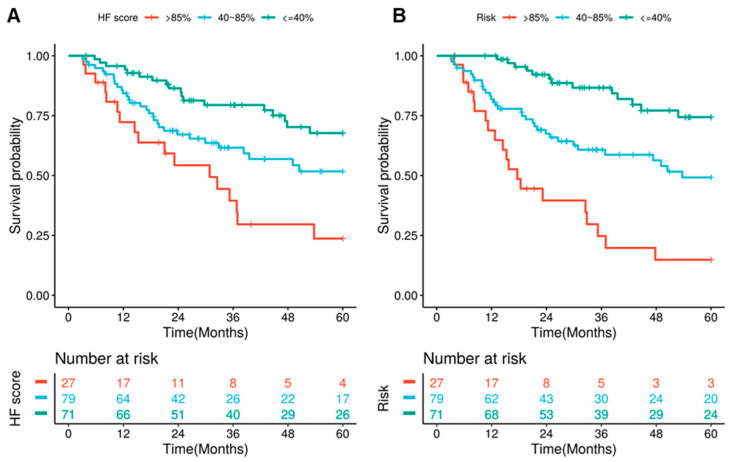
HF score and RSF prediction stratify the test cohort patients into distinct risk groups. The Kaplan–Meier estimates for the patient stratified based on the HF score (**A**) and risk predicted by RSF model (**B**) based on cutoffs of the 40th and 85th percentiles. The cutoffs were selected to be comparable with the H-index.

We further stratified the test cohort patients based on the 40th and 85th percentiles of the predicted mortality by RSF. The cutoff values were previously used by Valero et al. for the H-index they derived for the stratification of oral cavity cancer patients [5]. We used the same set of cutoffs for comparison purpose. Survival analysis showed that the group with highest HF scores has significantly higher risk than the low and middle groups in terms of five-year OS (Figure 4A, *p* < 0.0001). The hazard ratios (HRs) are 3.76 (95% CI:1.93–7.32) and 1.93 (1.07–3.50), respectively. The HRs are both slightly higher than that of the H-index (3.22 and 1.47) [5]. The HRs are further improved to 7.41 (3.66–14.98) and 2.58 (1.47–4.51) when stratified by the RSF predicted risk, which combined the HF score and clinical/demographic information (Figure 4B). It should be noted the higher HRs does not mean the HF score outperforms the H-index, as the underlying patient population is different. Instead, our results verified the finding that host factors are predictive of head and neck cancer patients’ OS.

## 4. Discussion

In this study, we used machine learning techniques to build a multivariate model for the prediction of OS in HNSCC patients receiving radiation therapy based on an institutional data set. We further evaluated the contribution of host factors in improving the prediction. The selected model was rigorously validated in test datasets separated before any processing and model training steps to ensure the model’s robustness.

The result shows that RSF achieved the best performance among all common modeling methods tested. The model achieved a C-index of 0.729, and with all AUCs above 0.7 for year 1~5. In particular, the AUC for two-year OS is 0.792, and over 0.8 for one-year OS. Notably, the model only relies on measurements that are routinely collected in clinical settings, so it is highly useful in practice. Our work recapitulated that a model that can handle nonlinear associations and complex interactions works better in predicting head and neck cancer patients’ OS. Indeed, nonlinear relationships between predictors and survival rates were observed. The deep learning model is capable of automatic feature extraction, so it may be able to learn the relationship among host factors. However, it did not show advantage in our study, which is possibly due to the moderate sample size available. Another contribution of this work is we verified the previous finding that host factors are independently associated with prognosis of head and neck cancer patients [5], and objectively assessed the improvement in prediction performance by their inclusion into the predictive models. To efficiently utilize the host factor information, we proposed the application of PCA. The first component naturally provides a composite score of the states of two groups of factors, which makes interpretation of this score biologically convenient. The HF score alone stratifies the HNSCC patients in the test cohort into distinct risk groups with excellent discriminating capacity. This finding also suggests that dimension reduction is crucial for efficient usage of host factor information by machine learning models, at least when the sample size is moderate.

It should be noted that we included different patient subgroups who underwent surgical treatment and received exclusive chemoradiation therapy. The tree based (RSF, RF, GBM) and neural network machine learning models (DeepSurv and ANN) are expected to account for the disparity in associations between predictors and outcomes in different subgroups. On the other hand, if the associations are shared among subgroups the strategy of combining cohorts is expected to boost the model performance. The C-indices for predicting OS in patients with definitive and post-operative radiotherapies are 0.746 (0.028) and 0.710 (0.081), respectively. Therefore, the prediction for non-surgical patients is slightly better, though we cannot accurately evaluate the accuracy in surgical patients due to the small sample size. In addition, some prognostic factors such as pathological grading were not found to be independently predictive in our work. This is possibly due to the fact that most patients had moderately and poor differentiated tumors (Table 1).

Recently, a convolutional neural network (CNN) applied to pre-treatment computed tomography (CT) images achieved an AUC of 0.7 for two-year OS, which was trained on a much smaller data set (*n* = 194) [26]. Cozzi et al. used CT-based radiomics and attained a C-index of 0.9 in predicting the OS locally advanced head and neck cancer patients. However, there were 40 patients in the validation cohort with only three events [27]. Similarly, a study reported a C-index of 0.781 for cancer-related death for oral cancer patients but there are only 11 events in the test cohort [28]. The model development and selection procedures were not described in detail, making it difficult to evaluate the model’s generalizability. In addition, the study used cancer recurrence as a predictor, which contributes roughly 0.06 to the prediction accuracy based on the VIMP reported. However, this is not applicable for primary cancer patients at baseline. In another study, Hung et al. used demographic and clinical data to model oral cancer survival as a continuous variable with all censored observations excluded [29]. The study was conducted on a patient cohort spanning from 1975 to 2016 and the year of diagnosis was found as the most predictive feature. Due to the high heterogeneity in diagnostic years and different metrics of performance, the model is not directly comparable with ours [29]. Moreover, the output of these models cannot be directly converted into survival functions or survival probabilities, so the application is quite limited. In another study, Howard et al. investigated the usage of machine learning in the guidance of the adjuvant treatment of head and neck cancers. While other models had better performance on personalized treatment recommendations, RSF showed best performance in predicting the OS with a C-index of 0.695 [23]. This observation is consistent with our results using clinical and demographic features alone (0.703). Other studies used machine learning models to predict different outcomes such as toxicities [30].

None of the above work investigated the roles of host factors in prognostic modeling. Valero et al. derived the H-index based on host factors for oral cavity cancer patients. While the authors did not assess the index’s discriminating ability, based on their categorization, the HR between the highest and lowest risk group (top 14.5% and bottom 38.6%) for OS is 3.22, which is lower than the HF score we derived [5]. Note that our results may not be comparable due to the difference in patient groups. However, our result verified that the host factors are independent predictors of head and neck cancer patients’ OS.

The study may have some intrinsic limitations due to its retrospective nature. In addition, the model was only internally validated. We are currently building a cohort for validation to ensure the model’s generalizability. Based on the stratification by our predictive model, we will be able to provide supportive care for patients likely to have worst outcome, and design trials specifically on the high-risk patients and test novel treatment management paradigms. As a next step, models predicting adverse events or toxicities such as kidney injury from Cisplatin will be investigated. Another important direction is to investigate whether host factor can further improve the prediction performance in combination with radiomics.

## 5. Conclusions

In this work, we evaluated common machine learning models in predicting the head HNSCC patients’ overall survival based on clinical, demographic features and host factors. We found the random survival forest had best performance among the models evaluated. We further verified that host factors are independently predictive of HNSCC overall survival and proper dimension reduction is an important step for their incorporation into the machine learning models. The score derived from this process also showed excellent discriminating capacity by itself. The results are of critical importance for improved risk stratification of HNSCC patients and providing targeted supportive care for patients who are likely to have worst outcome.

## Figures and Tables

**Table 1:** Summary of patient characteristics.

Variable	Value	Frequency/Median	Percentage/IQR	Total
Age		60.49	(54.22, 66.86)	591
BMI		27.09	(23.82, 30.24)	573
Weight loss		6	(2.5, 9.8)	565
Karnofsky Performance Status		9	(8,10)	591
Dose of primary radiotherapy		70	(70, 70)	587
Radiotherapy duration		46	(45, 46)	591
Gender	Male	483	81.7%	591
	Female	108	18.3%	
Marital status	Married	298	50.4%	591
	Other	293	49.6%	
Anti-coagulants	No	546	92.4%	591
	Yes	45	7.6%	
NSAIDs	No	322	54.5%	591
	Yes	269	45.5%	
Alcohol consumption	Never	105	18.6%	566
	Former	124	21.9%	
	Current	337	59.5%	
Smoking status	Never	131	22.2%	591
	Former	301	50.9%	
	Current	159	26.9%	
Site	Oral cavity/lip	67	11.3%	591
	Oropharynx	257	43.5%	
	Hypopharynx	43	7.3%	
	Nasopharynx	15	2.5%	
	Larynx	142	24.0%	
	Salivary gland	9	1.5%	
	Not specified	52	8.8%	
	Other	6	1%	
Clinical stage	I	18	3.1%	575
	II	46	8%	
	III	454	79%	
	IV	57	9.9%	
Pathological grading	Well differentiated	40	8.4%	
	Moderately differentiated	227	47.4%	
	Poorly differentiated	204	42.6%	
	Undifferentiated	8	1.7%	
HPV	Negative	131	39.3%	333
	Positive	202	60.7%	
Treatment type	RT only	33	5.6%	591
	CCRT	364	61.6%	
	Surgery + CCRT	106	17.9%	
	Surgery + RT	25	4.2%	
	CCRT + Neck Dissection	7	1.2%	
	ICT + CCRT	56	9.5%	
Primary chemotherapy type	Other or no chemotherapy	136	23%	591
	Cisplatin	455	77%	
Radiotherapy delayed	No	565	96.6%	585
	Yes	20	3.4%	
Type of radiation	Definitive	474	80.2%	591
	Post-operative (adjuvant)	117	19.8%	
Laterality of radiation	Unilateral	99	32.6%	304
	Bilateral	205	67.4%	
Feeding tube type	No	250	42.3%	591
	Yes	341	57.7%	
Hospitalized	No	463	78.5%	590
	Yes	127	21.5%	

**Table 2:** Summary of host factors.

Variable	*N*	Median	IQR
WBC	591	7.25	(6.05, 9.13)
HGB	591	13.5	(12.1, 14.75)
HCT	591	40.1	(36.4, 43.3)
RBC	591	4.44	(3.96, 4.81)
MCV	591	90.7	(87.1, 93.9)
MCH	590	30.7	(29.5, 31.9)
MCHC	591	33.8	(33, 34.4)
Neutrophil (%)	591	65.8	(59.15, 72.4)
Lymphocyte (%)	591	23.9	(18.6, 30)
Monocyte (%)	591	6.2	(5.1, 7.5)
Eosinophil (%)	591	2.5	(1.5, 3.6)
Basophil (%)	590	0.5	(0.4, 0.7)

**Table 3:** Model performance evaluated by the average C-index from cross-validations.

Model	Validation C-Index
RSFc	0.707 (0.032)
RSF-1	0.721 (0.013)
RSF-2	0.717 (0.013)
RSF-3	0.717 (0.009)
RSF-ALL	0.705 (0.015)
COXc	0.671 (0.042)
COX-1	0.690 (0.024)
COX-2	0.690 (0.024)
COX-3	0.690 (0.024)
COX-ALL	0.686 (0.021)

**Table 4:** Model test performance measured by C-index and AUC of two-year survival. The cutoffs were selected by maximizing the Youden index. For classification models, the C-index is obtained based on the predicted two-year survival probability.

Model	C-Index	AUC	Specificity	Sensitivity	PPV	NPV
RSF	0.729 (0.027)	0.792 (0.039)	0.615	0.864	0.487	0.914
RSFc	0.703 (0.029)	0.758 (0.042)	0.663	0.795	0.500	0.885
COX	0.679 (0.035)	0.718 (0.048)	0.817	0.568	0.568	0.817
COXc	0.636 (0.036)	0.712 (0.047)	0.808	0.545	0.545	0.808
DeepSurv	0.712 (0.029)	0.759 (0.042)	0.731	0.705	0.525	0.854
RF	0.719 (0.029)	0.771 (0.040)	0.625	0.818	0.480	0.89
Logistic	0.697 (0.035)	0.740 (0.050)	0.760	0.682	0.545	0.849
ANN	0.640 (0.037)	0.660 (0.050)	0.750	0.568	0.490	0.804
GBM	0.717 (0.030)	0.767 (0.040)	0.683	0.750	0.500	0.866

## Data Availability

The data underlying this article cannot be shared publicly for the privacy of individuals that participated in the study. The data are available from the corresponding author upon reasonable request.

## Supplementary Materials

The following are available online at https://www.mdpi.com/article/10.3390/cancers13184559/s1, File S1: Detailed infromation of Modeling Strategy.
