# Supplementary material for: Machine Learning Incorporating Host Factors for Predicting Survival in Head and Neck Squamous Cell Carcinoma Patients
Source: Cancers (Basel). 2021 Sep 11;13(18):4559. doi: 10.3390/cancers13184559 (PMC8467754; doi:10.3390/cancers13184559)
Supplement: Supplementary file 1 [file cancers-13-04559-s001.zip › cancers-1356999-supplementary.pdf]

cancers

MDPI

Supplementary Materials: Machine Learning Incorporating Host Factors for Predicting Survival in Head and Neck Squamous Cell Carcinoma Patients

Han Yu, Sung Jun Ma, Mark Farrugia, Austin J. Iovoli, Kimberly E. Wooten, Vishal Gupta, Ryan P. McSpadden, Moni A. Kuriakose, Michael R. Markiewicz, Jon M. Chan, Wesley L. Hicks, Jr., Mary E. Platek and Anurag K. Singh

Methods

Modeling Strategy

A three-fold cross-validation (CV) within the training/validation cohort was used for model selection. For each run, the hyperparameters of COX(LASSO) and GBM were tuned by a nested five-fold CVs. For each GBM model, a random hyperparameter search was performed with nested five-fold CVs. A hundred combinations of randomly generated tuning parameters were created, including learning rate (0.0001~1), minimum loss required for further partition (0.1~10), max depth of a tree (2~10), subsample ratio of variables when training each tree (0.5~1), minimum sum of instance weight (1~5) and subsample ratio of the training instances (0.5~1). An optimal model was selected based on the average of appropriate evaluation metrics (C-index or AUC) across CV runs. The selected model was then be applied to the test set and its prediction performance was assessed using C-index and the AUC of the ROCs at selected time points. For ANN, we empirically selected a model with three hidden layers and 16, 8, 4 hidden units for each layer. A logistic activation function was used. For DeepSurv, we started with the optimal model architecture and configuration reported by [20], which was obtained using a large train set (n=26,281). The second hidden layer was removed to scale down the model due to a much smaller training sample size in our study. Using the original model and further model reduction was found to negatively impact the model performance.

Model interpretation

Following the work by Ishwaran et al., the partial dependence function for a variable X evaluated at $X = x$ is

$$\tilde{f}(x) = \frac{1}{n}\sum_{i=1}^{n} \hat{f}(x, x_{i,0}), \quad (1)$$

where $x_{i,0}$ represents the value for all other variable other than X for individual i and $\hat{f}$ is the predicted outcome.
